# Influencing factors of Barthel index scores among the community-dwelling elderly in Hong Kong: a random intercept model

**DOI:** 10.1186/s12877-021-02422-4

**Published:** 2021-09-06

**Authors:** Hao Pan, Yang Zhao, Hailiang Wang, Xinyue Li, Eman Leung, Frank Chen, Javier Cabrera, Kwok Leung Tsui

**Affiliations:** 1 School of Data Science, City University of Hong Kong, Hong Kong, China; 2 School of Public Health (Shenzhen), Sun Yat-sen University, Shenzhen, China; 3 School of Design, The Hong Kong Polytechnic University, Hung Hom, Hong Kong, China; 4 School of Public Health & Primary Care, The Chinese University of Hong Kong, Hong Kong, China; 5 Department of Management Sciences, City University of Hong Kong, Hong Kong, China; 6 Department of Statistics, Rutgers University, New Brunswick, NJ USA; 7 Grado Department of Industrial and Systems Engineering, Virginia Polytechnic Institute and State University, Blacksburg, Virginia, USA

**Keywords:** Barthel index, Influencing factors, Electronic health records, Linear mixed effects model

## Abstract

**Background:**

Barthel Index (BI) is one of the most widely utilized tools for assessing functional independence in activities of daily living. Most existing BI studies used populations with specific diseases (e.g., Alzheimer’s and stroke) to test prognostic factors of BI scores; however, the generalization of these findings was limited when the target populations varied.

**Objectives:**

The aim of the present study was to utilize electronic health records (EHRs) and data mining techniques to develop a generic procedure for identifying prognostic factors that influence BI score changes among community-dwelling elderly.

**Methods:**

Longitudinal data were collected from 113 older adults (81 females; mean age = 84 years, SD = 6.9 years) in Hong Kong elderly care centers. Visualization technologies were used to align annual BI scores with individual EHRs chronologically. Linear mixed-effects (LME) regression was conducted to model longitudinal BI scores based on socio-demographics, disease conditions, and features extracted from EHRs.

**Results:**

The visualization presented a decline in BI scores changed by time and health history events. The LME model yielded a conditional R^2^ of 84%, a marginal R^2^ of 75%, and a Cohen’s f^2^ of 0.68 in the design of random intercepts for individual heterogeneity. Changes in BI scores were significantly influenced by a set of socio-demographics (i.e., sex, education, living arrangement, and hobbies), disease conditions (i.e., dementia and diabetes mellitus), and EHRs features (i.e., event counts in allergies, diagnoses, accidents, wounds, hospital admissions, injections, etc.).

**Conclusions:**

The proposed visualization approach and the LME model estimation can help to trace older adults’ BI score changes and identify the influencing factors. The constructed long-term surveillance system provides reference data in clinical practice and help healthcare providers manage the time, cost, data and human resources in community-dwelling settings.

## Background

Aging reduces older adults’ physical and cognitive capacities and further affects their basic activities of daily living (ADL) [1]. Long-term clinical surveillance of older adults’ functional independence in ADL has become necessary in community-based elderly care [2, 3]. Several reliable assessment scales have been developed for monitoring functional changes over time [4, 5]. The Barthel Index (BI) is one of the most commonly used scales for measuring functional independence status and especially for assessing improvements during rehabilitation [6]. The BI scale measures respondents’ capability in 10 activities (e.g., feeding, bathing, dressing, etc.), with a total score ranging from 0 to 100 [7]. Lower BI score is associated with greater future disability, longer time and greater care needs for recovery [8]. The BI scale had well-established validity and reliability, with Cohen *κ*, ranging from good (0.61–0.80) to very good (0.81–1.00), and internal consistency (Cronbach *α*), ranging from good (0.80–0.89) to excellent (0.93) [9, 10].

Previous studies have reported some factors affecting BI score changes in specific populations, such as patients with Alzheimer’s disease [11], heart failure [12], stroke [3, 6, 13–15], cancer, and tumor [16]. These studies presented that BI scores could be affected by heterogeneous demographic information (e.g. age, sex), measurement time [6, 17], and multiple prognostic factors (e.g. psychological factors and social support factors) [1, 14, 18]. However, in long-term surveillance, aging will bring in physical frailty and lead to a variety of health conditions that can affect older adults’ functional status and further affect their BI scores [4, 19]. Such heterogeneity limited the generalization of the results from prospective studies that only used one type of disease cohort.

To ensure the validity of the BI assessments in a general setting among community-dwelling elderly, it is important to choose the professional researcher, nurse, care giver, or therapist for the data collection. However, assessing the BI score at each occurrence of different diseases would increase the workload for healthcare providers in the practical service [20]. Moreover, the efficiency of the traditional BI calculation was limited, particularly for various populations with different diseases, e.g., speech disorders (including dysphasia), depression, or cognitive function, leading to insufficiently sensitivity. This issue could be addressed by using together with other scales/datasets in the long-term assessment [20], for example, time-varying electronic health records (EHRs) data that included possible important prognostic factors such as depression, medical comorbidities [6].

BI scores have usually been assessed annually and recorded in EHRs at nursing homes; meanwhile, EHRs are comprised of records of individual health history, such as diagnosis of disease, medical care, vaccine injections, length of stay in hospitals, etc. [21]. As the health history events occurred irregularly and unequally among individuals, the events in the EHRs provided different resolutions with the routinely assessed BI scores [22]. To fix these issues, integrated visualization is needed to pre-process the data and extract valuable information from individual EHRs [23]. Previous studies have been conducted to visualize different types of data (e.g., health events and durations in health history) following the timelines [24, 25]. However, the visualization between health histories in individual EHRs and the trajectory of the longitudinal BI scores was not well established. Further, the time varying features from aligned visualization of individual EHRs could depict the progression of health histories but have not been widely used in the association with BI scores [6].

Thus, the present study was designed to utilize EHRs and data mining techniques to develop a generic procedure for identifying prognostic factors that influence BI score changes among community-dwelling elderly. In addition, to visualize the individual EHRs and monitor the trajectories of BI scores, we extracted features from long-term EHRs and conducted statistical models to handle the heterogeneity among individuals. We utilized linear mixed-effects (LME) regression to examine whether annually assessed BI scores in geriatric residents were associated with socio-demographics, disease conditions, and extracted features in EHRs. We also compared the fitting performance of competitive models and used the modeling results to identify the factors that had statistically significant effects on the change of BI scores.

## Methods

### Datasets

This retrospective study included 113 participants aged 65 or above from Hong Kong elderly care centers. The EHRs of the participants between 06 August 2005 and 06 July 2016 were retrieved as the dataset. All the EHRs contained: a) more than one completed BI assessment period; b) more than two repeated BI scores; and c) additional records of socio-demographics and health histories.

The BI total score was utilized in the present study as it has a good (0.80–0.89) to excellent (0.93) internal consistency in previous studies (e.g., rehabilitation settings) [10, 20]. It ensured the full utilization of its numeric information [6] and overcome the ordinal non-hierarchical nature of the section scores in BI scales [20]. Registered nurses in the tested nursing homes performed the BI evaluation for each individual at various time points. The mean time between two BI assessments was 315.5 days, with the median as 336.0 days (IQR 301.0 to 340.0 days). A total of 605 observations of BI scores were included, covering the 492 assessment periods and with a mean of 5.4 assessment times (SD = ±1.9) per participant.

In the EHRs, the participants’ baseline characters were comprised of BI scores, socio-demographics (i.e., age, sex, marriage, religion, education, living arrangements, hobbies), and disease conditions (see Table 1). Health history in EHRs included allergy remarks, acute accidents, diagnosis of disease, revisit records, injection records, hospital admissions and discharges, medical cares, specialized nursing, wound care, and off-home records for periods away from the nursing home. We categorized the participants into two groups: the “active” cohorts, including 55 people who were alive at the end of the data collection time; and the “inactive” group, including 58 residents who left the nursing centers before the end of follow-up for specific reasons (e.g., died or moved to other facilities). This study was approved by the Research Ethics Committee of City University of Hong Kong (reference no.: 2–1-201510_01).

**Table 1:** Baseline characteristics

	Full cohort (*n* = 113)	Active cohort (*n* = 55)	Inactive cohort (*n* = 58)
BI scores (mean ± SD)	73.5 ± 29.3	78.0 ± 26.3	68.4 ± 31.7
Age (years) (mean ± SD)	84.3 ± 7.0	81.8 ± 6.7	86.7 ± 6.5
Male (n (%))	32 (28.3)	15 (27.3)	17 (29.3)
Marital status (n (%))			
Married	17 (15.0)	13 (23.6)	4 (6.9)
Divorced	12 (10.6)	9 (16.4)	3 (5.2)
Widowed	69 (61.1)	30 (54.6)	39 (67.2)
Single	13 (11.5)	2 (3.6)	11 (19.0)
NA	2 (1.8)	1 (1.8)	1 (1.7)
Religion (n (%))			
Buddhism	8 (7.1)	4 (7.3)	4 (6.9)
Catholic	2 (1.8)	1 (1.8)	1 (1.7)
Christian	7 (6.19)	2 (3.6)	5 (8.6)
Taoism	2 (1.8)	2 (3.6)	–
None	55 (48.7)	28 (50.9)	27 (23.9)
NA	39 (34.5)	18 (32.7)	21 (18.6)
Education (n (%))			
None	21 (18.6)	16 (29.1)	5 (8.6)
Primary	11 (9.7)	9 (16.4)	2 (3.4)
Secondary	2 (1.8)	2 (3.6)	0 (0)
Junior High	33 (29.2)	11 (20.0)	22 (46.6)
Senior High	24 (21.2)	6 (10.9)	18 (31.0)
College	1 (0.9)	1 (1.8)	–
Graduated University	1 (0.9)	1 (1.8)	–
NA	20 (17.7)	9 (16.4)	11 (19.0)
Living arrangement (n (%))			
Living with spouse	4 (3.5)	3 (5.5)	1 (1.7)
Living with children	23 (20.4)	13 (23.6)	10 (17.2)
Living with other relatives	5 (4.4)	2 (3.6)	3 (5.2)
Living with others	43 (38.1)	23 (41.8)	20 (34.5)
Living alone	22 (19.5)	8 (14.6)	14 (24.1)
NA	16 (14.2)	6 (10.9)	10 (17.2)
Hobby (n (%))			
Travel	26 (23.0)	13 (23.6)	13 (22.4)
Reading	19 (16.8)	11 (20.0)	8 (13.8)
Partying	62 (54.9)	21 (38.2)	41 (36.28)
Outside	64 (56.6)	31 (56.4)	33 (29.2)
Mahjong	13 (11.5)	7 (12.7)	6 (10.3)
TV	70 (62.0)	33 (60.0)	37 (22.4)
Teamwork	34 (30.1)	14 (25.5)	20 (34.5)
Gambling	8 (7.1)	3 (5.5)	5 (8.6)
Sports	21 (18.6)	10 (18.2)	11 (19.0)
Smoking	10 (8.9)	2 (3.64)	8 (7.08)
Weaving	3 (2.7)	2 (3.64)	1 (1.7)
Disease (n (%))			
Hypertension	83 (73.5)	42 (76.4)	41 (70.7)
Cataract	61 (54.0)	28 (50.9)	33 (56.9)
Dementia	43 (38.1)	13 (23.6)	30 (51.7)
Diabetes Mellitus	39 (34.5)	19 (34.6)	20 (34.5)
Anaemia	36 (31.9)	8 (14.6)	28 (48.3)
Pneumonia	33 (29.2)	13 (23.6)	20 (34.5)
Urinary Tract Infection (UTI)	21 (18.6)	8 (14.6)	13 (22.4)
Stroke	16 (14.2)	11 (20.0)	5 (8.6)
Cerebrovascular Accident (CVA)	14 (12.4)	6 (10.9)	8 (13.8)
Atrial Fibrillation (AF)	14 (12.4)	6 (10.9)	8 (13.8)
Depression	13 (11.5)	6 (10.9)	7 (12.1)
Heart Failure	13 (11.5)	1 (1.8)	12 (20.7)
Gastritis	12 (10.6)	4 (7.3)	8 (13.8)
Parkinson	9 (8.0)	3 (5.5)	6 (10.3)
Alzheimer	8 (7.1)	2 (3.6)	6 (10.3)
Traumatic	6 (5.3)	3 (5.5)	3 (5.2)
Sclerosis	2 (1.8)	1 (1.8)	1 (1.7)

### Data processing

The EHRs were restructured to aggregate various types of individual-linked data [26], which yielded flexible formulae matching the needs and authorities of specific retrospective cohorts [27]. In structured EHRs, individual health histories were captured in chronological order based on the individual assessment period of BI scores. The utilization and application of the structured EHRs lay in two directions: first, for the individual visualization, we employed the integrated plots to align the longitudinal BI scores with the acute events in health histories chronologically; second, for the influencing factor detection, we implemented the LME model to depict the association between longitudinal BI scores and prognostic factors [6], including socio-demographics, disease conditions, and extracted features from EHRs.

Seventeen disease conditions were retrieved from the diagnosis records in EHRs. The diseases with the top frequency of occurrence rate (above 10%) were hypertension, cataract, dementia, diabetes mellitus, anaemia, pneumonia, urinary tract infection, stroke, cerebrovascular accident (CVA), atrial fibrillation (AF), depression, and heart failure. Other diseases that were relevant to the change of BI scores were also listed, including Parkinson [19], Alzheimer [28], traumatic [29], sclerosis [30, 31], and epilepsy [32].

The extracted features from EHRs included 20 variables from observational records in health histories. At each assessment period of BI scores, we calculated the count of occurrences for time-to-event data and calculated the length of time for the duration data [33, 34]. The count of time-to-event data came from observational records regarding allergies, accidents, diagnoses, medicines, special medical cares, hospital admissions, injections, revisits, and off-home. The duration data described the length of stay in hospitals and the length of dates in taking medicines. Additional 9 features were the counts of the vaccine injection records.

### Data analysis

Univariate analysis was conducted to compare the BI scores among disease groups and a significant difference in BI score indicated heterogeneities among individuals. Student’s unpaired t-test was used to detect significant disease conditions in the group mean BI scores.

A linear mixed effect (LME) model with random intercepts was used to capture the heteroscedasticity for individuals [6, 35], and obtain the fixed effects to interpret variables of 46 prognostic factors. The full inclusions of the covariates in the LME model were 17 socio-demographics, 9 disease conditions with the significant difference in the Student’s unpaired t-test, and 20 health history features from structured EHRs.

To depict the correlation between different kinds of prognostic factors and BI scores, we implemented the stepwise regression in a linear model (LM) versus the LME model. We adopted several goodness-of-fit criteria, including R^2^, AIC, BIC, and log-likelihood, to evaluate the model performance. A consistent result in LM and LME models showed that the full inclusion of covariates yielded the best performance.

To identify the factors influencing the change of BI scores, we conducted a *χ*^2^ test with corresponding *p* values for the full inclusion of prognostic factors in the LME model. A summary of the significant effects in the LME coefficients was provided to determine the influencing factors of BI score changes. All analyses were carried out using the R program (version 3.6.2). The LME model was implemented with the “lmer()” function in “lme4” package [36]. Significance was set at *p* < 0.05.

## Results

### Visualization of BI scores and health events

**Fig. 1:**
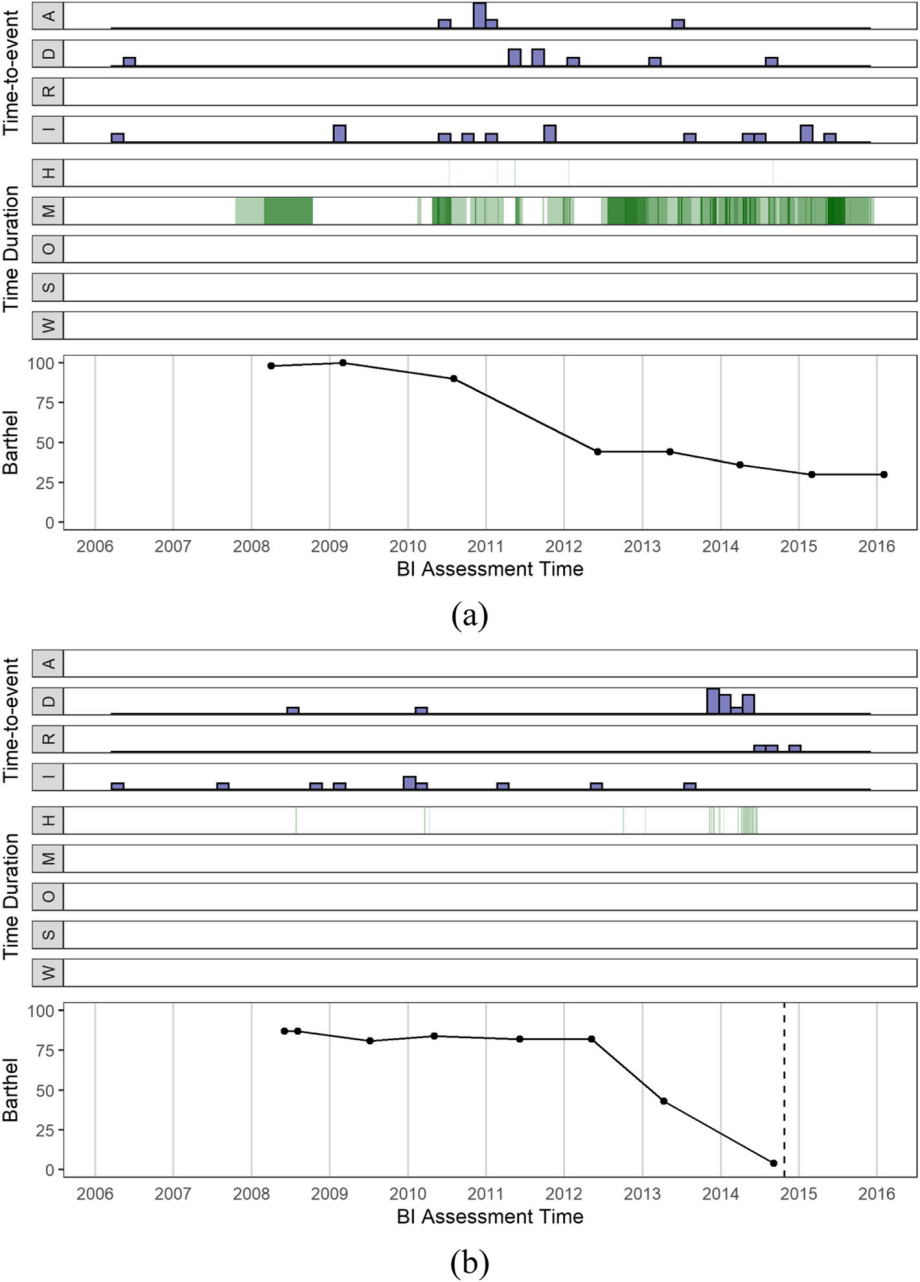
Individual visualizations for (**a**) one participant in the “active” group (top) and (**b**) one participant in the “inactive” group (bottom). The end of the follow-up period in BI scores were marked by a dashed line in (**b**). (A: “accidents”; D: “diagnosis”; R: “revisits”; I: “injections”; H: “hospital admissions”; M: “medical cares”; O: “off-home”; S: “special nursing”; and W: “wounds”)

Figure 1 shows the developed visualization approach by aligning individuals’ EHRs with their BI scores in a longitudinal way. In details, the occurrence of time-to-event data was presented in the top four bar-plot panels, with the bar height denoting the event counts. The following were the time-duration panels, with the width and the depth of the color representing the duration and frequency of health history events, respectively.

### Univariate analysis in disease groups

Table 2 shows the results of the Student’s unpaired t-test for group means of BI scores. There were significant differences in BI scores in the cohorts of cataract, dementia, diabetes mellitus, anaemia, pneumonia, urinary tract infection, Parkinson, Alzheimer, and sclerosis (all *p* values < 0.05).

**Table 2:** Univariate analysis of the BI records in disease groups

	BI Mean Disease	BI Mean Non-Disease	Df	t test	*P* value
Hypertension	73.7	72.6	273.4	0.4	0.68
Cataract	76.3	69.8	557.3	2.7	0.01*
Dementia	62.4	80.3	374.1	−7	< 0.001*
Diabetes Mellitus	78.1	71.2	496.8	3	< 0.001*
Anaemia	69.1	75.4	343.9	−2.4	0.02*
Pneumonia	68.8	75.2	267.5	−2.3	0.02*
Urinary Tract Infection	66.8	74.8	139.9	−2.5	0.01*
Stroke	67.5	74.3	88.5	−1.7	0.09
Cerebrovascular Accident	75.9	73.1	116.2	0.9	0.38
Atrial Fibrillation	69.8	73.9	86.6	−1.1	0.26
Depression	74.5	73.3	100.1	0.3	0.74
Heart Failure	71.1	73.8	93	−0.8	0.44
Gastritis	71.8	73.7	100.4	−0.5	0.6
Parkinson	57.1	74.6	45.6	−3.6	< 0.001*
Alzheimer	51.9	75.1	45.2	−3.8	< 0.001*
Traumatic	75.9	73.3	38.4	0.5	0.61
Sclerosis	97.1	72.8	26.1	9.9	< 0.001*

### Model comparisons and sensitivity analysis

Table 3 presents the comparison of the stepwise regression of covariates in LM and LME models. In Model-6, the full inclusion of the variables, including socio-demographics, disease conditions, and extracted features in EHRs, yielded the best performance according to all terms of the evaluation criteria. Comparing with Model-3 in the LM model, the formula of Model-6 in the LME model obtained a marginal R^2^ of 0.75 with fixed effects and a conditional R^2^ of 0.84 including individual random effects. When including EHR features, the Model-6 yielded a Cohen’s f^2^ of 0.68, which indicates a large effect size according to Cohen’s (1988) guidelines [37].

**Table 3:** Model comparison results of evaluation criteria

	**Formula**	**Random Effects**	**Multi.R**^**2**^	**Adj.R**^**2**^	**AIC**	**BIC**	**-Loglik**
LM	Model 1: BI ~ Demo	None	0.46	0.40	2519	2631	1229
	Model 2: BI ~ Demo + Disease		0.56	0.49	2481	2626	1201
	Model 3: BI ~ Demo + Disease + Health History		0.76	0.69	2360	2577	1120
	**Formula**	**Random Effects**	**Cond.R**^**2**^	**Marg.R**^**2**^	**AIC**	**BIC**	**-Loglik**
LME	Model 4: BI ~ Demo	ID	0.79	0.49	2433	2549	1185
	Model 5: BI ~ Demo + Disease		0.77	0.58	2432	2580	1175
	Model 6: BI ~ Demo + Disease + Health History		0.84	0.75	2337	2557	1107

Note: Demo represents 17 socio-demographic variables; Disease represented 9 disease conditions, Health History represented 20 extracted features from health history in EHRs. Multi.R^2^, multiple R square. Adj.R^2^, adjusted R square. Cond.R^2^, Conditional R square. Marg.R^2^, Marginal R square,

### Influential factors identified by LME models

Table 4 showed the results of the LME model with the full inclusion of covariates. The random intercepts were among individuals and the fixed effects included socio-demographics, disease conditions, and extracted features in EHRs. The baseline of BI score was 80.6 (SD = 30.3). The Chi-square test results showed that the following factors with significant influence on BI score changes: socio-demographics (i.e., sex, education, living arrangement, hobbies of partying and watching the TV), disease conditions (i.e., dementia and diabetes mellitus), and health history features (i.e., counts of events in allergies, diagnoses, accidents, wounds, hospital admissions, and vaccine injection of pneumococcal and panenza) (all *p* values < 0.05).

**Table 4:** Coefficients in LME models with significance test results

	(Intercept)	Est.	SD	Chisq	*P* value
		80.6	30.3		
Age	Age	−0.5	0.4	1.8	0.18
Sex	Male	19.5	7.3	7.2	0.01*
Marital Status	Married	−5.5	9.9	2.3	0.52
	Single	−7.4	11.4		
	Widowed	2.2	9.0		
Religion	Catholic	11.3	20.7	8.9	0.06
	Christian	−23.6	13.2		
	None	−12.7	11.1		
	Taoism	18.0	31.1		
Education	Junior High	41.8	27.3	40.9	< 0.001*
	None	4.6	26.3		
	Primary	9.8	24.6		
	Secondary	−59.4	30.3		
	Senior High	10.8	24.4		
Living Arrangement	Living with others	10.3	9.9	11.5	0.02*
	Living with children	14.4	10.8		
	Living with spouse	49.2	18.0		
	Living alone	18.4	9.2		
Hobby	Travel	2.4	9.9	0.1	0.81
	Reading	2.9	7.5	0.1	0.70
	Partying	17.3	8.0	4.7	0.03*
	Outside	−4.3	6.0	0.5	0.48
	Mahjong	1.3	7.5	0.0	0.87
	TV	−16.5	5.4	9.4	< 0.001*
	Teamwork	−12.0	13.0	0.9	0.36
	Gambling	2.9	14.9	0.0	0.84
	Sports	−8.7	15.5	0.3	0.58
	Smoking	−4.9	8.4	0.3	0.57
	Weaving	−6.8	10.9	0.4	0.54
Disease Condition	Cataract	2.3	6.4	0.1	0.72
	Dementia	−22.4	6.7	11.2	< 0.001*
	Diabetes Mellitus	16.8	6.9	6.0	0.01*
	Anaemia	−5.9	6.7	0.8	0.37
	Pneumonia	5.3	5.5	0.9	0.34
	Urinary Tract Infection	0.3	8.7	0.0	0.97
	Parkinson	−6.8	11.8	0.3	0.56
	Alzheimer	1.5	10.9	0.0	0.89
	Sclerosis	−12.9	18.0	0.5	0.47
Extracted Features in EHRs	Allergy Counts	−26.8	6.3	17.9	< 0.001*
	Diagnosis Counts	−3.2	0.5	39.3	< 0.001*
	Accident Counts	−2.8	1.3	4.8	0.03*
	Hospital Time Durations	−36.5	31.4	1.3	0.25
	Hospital Counts	1.5	0.6	5.4	0.02*
	Medicine Time Durations	1.4	1.0	1.9	0.17
	Medicine Counts	0.0	0.1	0.6	0.45
	Off-home Counts	0.2	0.2	1.6	0.21
	Wound Counts	−9.2	2.4	14.8	< 0.001*
	Special Nursing Counts	−13.9	8.7	2.6	0.11
	Injection Counts	−0.2	0.8	0.0	0.83
	Injection Fluvax Counts	−1.9	3.6	0.3	0.60
	Injection Vaxigrip Counts	−1.3	1.4	1.0	0.33
	Injection Pneumo Counts	−9.4	3.7	6.3	0.01*
	Injection Swine Flu Counts	−8.1	4.3	3.6	0.06
	Injection Fluarix Counts	1.0	2.0	0.2	0.63
	Injection ATT Counts	−0.7	1.7	0.2	0.68
	Injection VIT.B12 Counts	0.2	0.9	0.1	0.81
	Injection Prevenar 13 Counts	−4.3	14.7	0.1	0.77
	Injection Panenza Counts	33.6	14.1	5.6	0.02*

### Magnitude of significant effects

The results showed significant effects of socio-demographics, including sex, education, living arrangement, hobbies of partying and watching the TV, on BI score changes. In terms of BI score, males had an average of 19.5 points (SD = 7.3, *p* = 0.01) larger than females. Education status (*p* < 0.001) and living arrangements (*p* = 0.02) showed significant effects on individuals’ BI scores. Individual who had hobbies of partying and watching the TV had 17.3 points (SD = 8.0, *p* = 0.03) increased and 16.5 points (SD = 5.4, *p* = < 0.001) decreased on the BI scores separately.

For disease conditions (see Table 2), we found that dementia and diabetes mellitus showed a significant effect of − 22.4 points (SD = 6.7, *p* = < 0.001) and 16.8 points (SD = 6.9, *p* = 0.01) on BI scores separately.

There were negative effects of health history features, which included counts of events in allergies, diagnoses, accidents, and wounds. Over 90% of cases in the allergy events belonged to drug allergy, and remaining cases were from food allergens. Generally, an allergy event reported 26.8 points’ (SD = 6.3, *p* = < 0.001) decrease in the BI scores. Over 96% of accident records were fall events. An averaged accident event caused 2.8 points (SD = 1.3, *p* = 0.03) decline in the total BI scores. The diagnosis events and wound events were recorded according to the follow-up time. With per count increased, the diagnosis events and wound events had − 3.2 points (SD = 0.5, *p* = < 0.001) and − 9.2 points (SD = 2.4, *p* = < 0.001) changes on the BI scores, separately.

An increased count in the hospital events, containing a period with admissions and discharges, had a positive effect on BI score changes, with an average of 1.5 points (SD = 0.6, *p* = 0.02) increased. Individuals vaccinated with PANENZA for the pandemic A/H1N1 influenza outbreak had an average of 33.6 points (SD = 14.1, *p* = 0.02) increased in the BI score changes. Over 68% individuals had a regular injection of the Pneumo vaccine, showing a negative effect of 9.4 points (SD = 3.7, *p* = 0.01) decrease on the BI score changes over time.

## Discussion

We integrated EHRs and data mining techniques to detect the influencing factors of BI score changes among the community-dwelling elderly in Hong Kong. Our visualization provided an effective way to display the longitudinal BI scores with the individual health history. The influencing factors of BI score changes included socio-demographics (i.e., sex, education, living arrangement, and hobbies), disease conditions (i.e., dementia and diabetes mellitus), and the extracted features in EHRs (i.e., event counts in allergies, diagnoses, accidents, wounds, hospital admissions, injections, etc.).

The contributions are threefold: 1) the present findings fulfill the research gap of data mining in EHRs and study of longitudinal BI scores; 2) our study highlights the association between features extracted from EHRs and the BI score changes; 3) the LME model is able to support timely detection of the causes of BI score changes. The presented approaches can be generalized to other data that have similar structures as we utilized herein.

### Visualization

In the visualization plots (see Fig. 1), we examined the patterns of health events to understand the clinical features that affected BI scores. Notable increments in the counts of accidents and diagnoses of disease were found as BI scores decreased. Additionally, in the assessment period with lower BI scores, the duration of medical care and hospital admissions were longer than that in the period with higher BI scores. In line with the observed association in the visualizations, features such as the event count in accidents, diagnosis, and hospital admissions were also identified as influencing factors of BI score changes in the LME model (Table 4).

When applying individual EHRs into long-term functional assessment, the declines in BI score trajectories may be due to different effects from the features in EHRs, and further be affected by the sub-groups in age, socio-demographics, and disease conditions. The trajectories of declines in BI scores could be described by the progression of individual EHRs. Once we identified the influencing factors from the features in EHRs, we can predict the functional status from the different aspects in individual EHRs, scheduling additional and necessary care for the recovery [8, 20]. Similar insights were also employed in the comprehensive assessment system in hospital settings. Indeed, this technique has been used previously to demonstrate the association of cognitive decline in healthy older adults [22, 38].

### Influencing factors

For socio-demographics, there was a significant gender effect on BI scores, i.e., females had much lower BI scores than males, consistent with previous studies [4–6]. One possible reason was that in our present study, the female group had relative larger age (mean = 83.6) than the male group (mean = 82.8) at the first time of BI score assessment (i.e., age at study entry). It indicates that our female subjects’ physical capability might be worse than that of males, leading to poorer BI performance. Our results showed non-significant effect of age in the LME model with an inclusion of EHR features. On the one hand, during the follow-up period, the functional status declined with age naturally in older adults [6], which led to the trajectory of BI scores decreasing gradually over time. On the other hand, the functional status might be changed during an acute event for each individual. The EHR features, such as disease progression, hospitalization, and medical care dominated the change of BI scores at that period. Thus, when including the EHR features in the LME model, the age is not significant in both unadjusted and adjusted models. In addition, we showed that education and living arrangements had significant effects on the change of BI scores. These two variables were commonly used in the prediction of BI scores [11] and functional dependence [3]. Moreover, older adults with hobby “partying” had significant positive effects on BI scores, while groups with hobby “TV” showed significant negative effects. This might be due to that people in the party were more likely to improve their performance in ADL, further preventing their BI scores from deteriorating.

For disease conditions, it is worth mentioning that our significant negative effect of dementia was consistent with previous studies [5, 39]. A short-term follow-up study showed that the BI scores had significantly different values in patients with or without dementia during hospitalization and after discharge [39]. For another significant disease, Murcia et al. (2010) supported that the distribution of diabetes mellitus had significant differences according to BI scores [5]. For EHR features, it was likely that older adults at frailty are vulnerable to acute events [4, 19]. Over 96% of accident records were fall events. It has been shown that people with low BI scores were identified as a high-risk group for falling during an in-patient stay [28]. Our study revealed such an association in both in-patient and out-patient periods, including older adults with higher BI scores. Moreover, older adults who had diagnosis records were identified as high-risk groups with certain diseases, such as infection, heart disease, and dehydration [4, 19]. Consequently, significant influencing factors in extracted features of EHRs also act as explanatory variables for monitoring the BI score changes, such as progression of allergies, wounds, diseases. Thus, using the counts of allergies, wounds, and diagnosis to monitor the change of BI scores is appropriate in community-dwelling older adults with different disease conditions.

### The LME model

The stepwise procedure and model comparison analysis showed the strengths of full inclusion variables in the LME model. Unlike the categorical variables in demographics and disease conditions, which affected the baseline of BI scores in different subgroups, health history features in EHRs varied along with time, influencing the BI scores chronologically. The inclusion of features in EHRs significantly improved the modeling likelihood in both LM and LME models. Furthermore, the employed random effect of ID information enhanced the interpretation ability of individual BI scores in the LME model, which was superior to the original least squares. The LME approach takes account of the correlation between repeated measurements on the same individual. It is also used in a short-term study of regular assessed longitudinal BI scores [6].

Based on the significant association between the BI scores and EHR features, there are two aspects for the practical use of the accurate prediction of BI scores, sequentially obtained in the current and the next assessment period. In the LME approach, the EHR features acted as novel adjustments, and the BI estimates are more accurate over a wide range of different disease groups. For instance, even in the same subgroup with specific socio-demographical variables and disease conditions, each point increase in the diagnosis counts might diminish 3 points decrease in the expected (or estimated) annual BI scores. Individuals with different value of the EHR features will receive different care services. Moreover, a period with increasing EHR features might report a severe change of the BI scores. This information implies an additional requirement of the BI assessment at that period. The surveillance system allows the management of time, cost, data, and people in the care teams.

The strengths of our study lie in two application scenarios. In hospital settings, as the BI scores decreased with increasing age gradually, a steep decline of BI scores for the patient was highly associated with an acute event. Consequently, incorporating BI scores in individual EHRs in both inpatient and outpatient cares improves the services in the clinical practice. In community settings, the BI scores were assessed routinely in the annual or bi-annual periods. A significant decline of BI scores indicates severe frailty in personal functional status at that period. In this circumstance, change of the BI scores over the assessment period may be a powerful predictive tool for decreased abilities for activities of daily living, which call for intervention to provide greater care and facilitate recovery [20].

There were some limitations in the present study. First, our findings were derived from one population, i.e., Hong Kong older adults in nursing homes. It remains unclear whether the results can be generalized to other populations, which merits more studies. Even so, the visualization approach that we developed can help to visualize the datasets with similar structure as we utilized, regardless of the study population. Second, the number of participants in our study was relatively small, although the LME model handles the heterogeneity of longitudinal BI scores well for even smaller study groups. As the LME model makes full use of the data, in future work, we could harness a larger sample size in other regions, and we can potentially consider more covariates such as regional or local factors without dimensionality problems. Third, we did not examine inter-observer variations of the longitudinal BI scores. However, previous studies reported the Barthel scale with good (κ = 0.62) to near perfect (κ = 0.99) inter-observer reliability with sample size ranging from 55 to 122 [9, 20].

## Conclusions

The present study proposed a visualization approach to correlate individual EHRs with BI scores chronologically. The LME model revealed some influencing factors for BI score changes from the perspectives of socio-demographics, disease conditions and extracted features in EHRs, among a sample of community-dwelling elderly in Hong Kong. The present findings could provide reference data on BI to facilitate elderly care providers in practical decision making and early interventions.

## Data Availability

The datasets generated and/or analyzed during the current study are not publicly available due to Institutional Review Board related matters but are available from the corresponding author on reasonable request.

## Abbreviations

BI: Barthel Index
EHRs: Electronic health records
LME: Linear mixed-effects
UTI: Urinary tract infection
CVA: Cerebrovascular accident
AF: Atrial fibrillation
